# *catena*-Poly[[bis­(3-methyl­benzoato-κ^2^ *O*,*O*′)lead(II)]-μ-4,4′-bipyridine-κ^2^ *N*:*N*′]

**DOI:** 10.1107/S1600536811035021

**Published:** 2011-09-03

**Authors:** Jian-Ying Xie, Fu Huang

**Affiliations:** a College of Science, Guangdong Ocean University, Zhanjiang 524088, People’s Republic of China

## Abstract

In the title complex, [Pb(C_8_H_7_O_2_)_2_(C_10_H_8_N_2_)]_*n*_, the Pb^II^ atom is located on a twofold rotation axis and is six-coordinated by four carboxyl­ate O atoms from two 3-methyl­benzoate ligands and two N atoms from two 4,4′-bipyridine (4,4′-bpy) ligands, displaying a hemi-directed coordination. The 4,4′-bpy ligand has an inversion center at the mid-point of the central C—C bond. The Pb^II^ atoms are linked by bidentate bridging 4,4′-bpy into a chain along [101]. These chains are further connected into layers *via* C—H⋯O hydrogen bonds.

## Related literature

For general background to 3-methyl­benzoate complexes, see: Wang *et al.* (2002 ▶); Zhao *et al.* (2009 ▶) and to 4,4′-bipyridine complexes, see: Biradha *et al.* (2006 ▶). For hemi- and holo-directed geometries of lead(II) complexes, see: Shimoni-Livny *et al.* (1998 ▶).
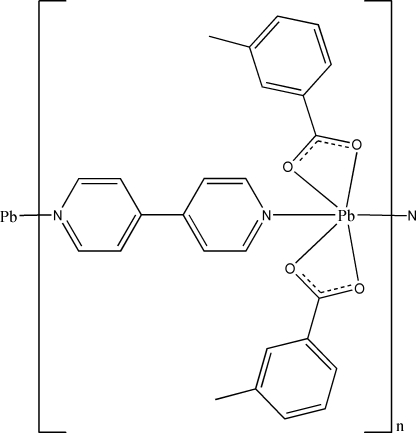


## Experimental

### 

#### Crystal data

[Pb(C_8_H_7_O_2_)_2_(C_10_H_8_N_2_)]
*M* *_r_* = 633.65
Monoclinic, 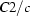

*a* = 20.506 (8) Å
*b* = 5.534 (2) Å
*c* = 20.219 (8) Å
β = 103.507 (7)°
*V* = 2231.0 (15) Å^3^
*Z* = 4
Mo *K*α radiation
μ = 7.60 mm^−1^
*T* = 296 K
0.30 × 0.27 × 0.21 mm

#### Data collection

Bruker APEXII CCD diffractometer
Absorption correction: multi-scan (*SADABS*; Sheldrick, 1996 ▶) *T*_min_ = 0.129, *T*_max_ = 0.215
8381 measured reflections
2402 independent reflections
2153 reflections with *I* > 2σ(*I*)
*R*_int_ = 0.025

#### Refinement

*R*[*F* ^2^ > 2σ(*F* ^2^)] = 0.017
*wR*(*F* ^2^) = 0.042
*S* = 1.01
2402 reflections
151 parameters
H-atom parameters constrained
Δρ_max_ = 0.38 e Å^−3^
Δρ_min_ = −0.50 e Å^−3^

### 

Data collection: *APEX2* (Bruker, 2007 ▶); cell refinement: *SAINT* (Bruker, 2007 ▶); data reduction: *SAINT*; program(s) used to solve structure: *SHELXS97* (Sheldrick, 2008 ▶); program(s) used to refine structure: *SHELXL97* (Sheldrick, 2008 ▶); molecular graphics: *SHELXTL* (Sheldrick, 2008 ▶) and *DIAMOND* (Brandenburg, 1999 ▶); software used to prepare material for publication: *SHELXTL*.

## Supplementary Material

Crystal structure: contains datablock(s) I, global. DOI: 10.1107/S1600536811035021/hy2458sup1.cif

Structure factors: contains datablock(s) I. DOI: 10.1107/S1600536811035021/hy2458Isup2.hkl

Additional supplementary materials:  crystallographic information; 3D view; checkCIF report

## Figures and Tables

**Table 1:** Selected bond lengths (Å)

Pb1—O1	2.4803 (19)
Pb1—O2	2.4148 (19)
Pb1—N1	2.893 (2)

**Table 2:** Hydrogen-bond geometry (Å, °)

*D*—H⋯*A*	*D*—H	H⋯*A*	*D*⋯*A*	*D*—H⋯*A*
C13—H13⋯O1^i^	0.93	2.54	3.461 (4)	172

Symmetry code: (i) 
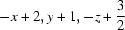
.

**Fig. 1.**
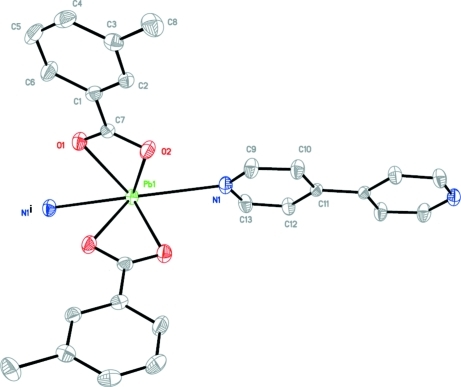
The asymmetric unit of the title compound. Displacement ellipsoids are drawn at the 30% probability level. H atoms have been omitted for clarity. [Symmetry codes: (i) 2-x, y, 3/2-z; (ii) 3/2-x, 3/2-y, 1-z.]

**Fig. 2.**
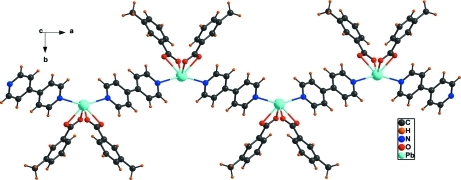
View of the chain in the title compound.

**Fig. 3.**
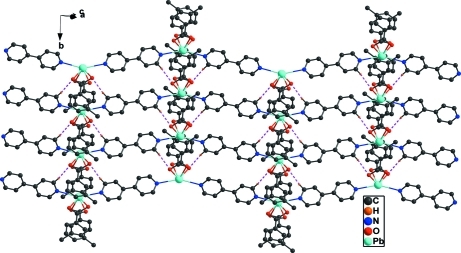
View of the layered network in the title compound. C—H···O hydrogen bonds are shown as dashed lines. H atoms not involved in hydrogen bonds have been excluded for clarity.

## supplementary crystallographic information

### Comment

In the structural investigation of 3-methylbenzoate complexes, it has been found that 3-methylbenzoic acid functions as a multidentate ligand (Wang *et al.*, 2002; Zhao *et al.*, 2009), with versatile binding and coordination modes. As is well known, 4,4'-bipyridine (4,4'-bpy) ligand may act in bidentate bridging or monodentate terminal mode (Biradha *et al.*, 2006). In this paper, we report the crystal structure of the title compound, a new Pb(II) complex obtained by the reaction of 3-methylbenzoic acid, 4,4'-bpy and lead acetate in an alkaline aqueous solution.

As depicted in Fig. 1, the Pb^II^ atom is located on a twofold rotation axis and is coordinated by four O atoms from two 3-methylbenzoate ligands and two N atoms from two µ-4,4'-bpy ligand (Table 1). The coordination environment of the Pb^II^ atom is hemidirected (Shimoni-Livny *et al.*, 1998). The 3-methylbenzoate ligand adopting bidentate coordination mode chelate the Pb^II^ atom, which can be regarded as a knot. The 4,4'-bpy ligand bridges two neighboring knots, forming a one-dimensional chain along [1 0 1] (Fig. 2). The distance between two knots is 12.882 (3) Å. These chains are further assembled *via* C—H···O hydrogen bonds (Table 2) into a layered network (Fig. 3).

### Experimental

A mixture of lead acetate (1 mmol, 0.325 g), 3-methylbenzoic acid (1 mmol, 0.136 g), 4,4'-bpy (1 mmol, 0.156 g), NaOH (1.5 mmol, 0.06 g) and H_2_O (12 ml) was placed in a 23 ml Teflon-lined reactor, which was heated to 433 K for 3 days and then cooled to room temperature at a rate of 10 K h^-1^. Colorless crystals obtained were washed with water and dried in air.

### Refinement

H atoms were placed at calculated positions and were treated as riding on the parent C atoms, with C—H = 0.93 (CH) and 0.96 (CH_3_) Å and with *U*_iso_(H) = 1.2(1.5 for methyl)*U*_eq_(C).

### Crystal data

**Table:**

[Pb(C_8_H_7_O_2_)_2_(C_10_H_8_N_2_)]	*F*(000) = 1224
*M**_r_* = 633.65	*D*_x_ = 1.887 Mg m^−^^3^
Monoclinic, *C*2/*c*	Mo *K*α radiation, λ = 0.71073 Å
Hall symbol: -C 2yc	Cell parameters from 5300 reflections
*a* = 20.506 (8) Å	θ = 1.3–28.0°
*b* = 5.534 (2) Å	µ = 7.60 mm^−^^1^
*c* = 20.219 (8) Å	*T* = 296 K
β = 103.507 (7)°	Block, colorless
*V* = 2231.0 (15) Å^3^	0.30 × 0.27 × 0.21 mm
*Z* = 4	

### Data collection

**Table:**

Bruker APEXII CCD diffractometer	2402 independent reflections
Radiation source: fine-focus sealed tube	2153 reflections with *I* > 2σ(*I*)
graphite	*R*_int_ = 0.025
φ and ω scans	θ_max_ = 27.0°, θ_min_ = 3.9°
Absorption correction: multi-scan (*SADABS*; Sheldrick, 1996)	*h* = −26→25
*T*_min_ = 0.129, *T*_max_ = 0.215	*k* = −7→2
8381 measured reflections	*l* = −25→25

### Refinement

**Table:**

Refinement on *F*^2^	Primary atom site location: structure-invariant direct methods
Least-squares matrix: full	Secondary atom site location: difference Fourier map
*R*[*F*^2^ > 2σ(*F*^2^)] = 0.017	Hydrogen site location: inferred from neighbouring sites
*wR*(*F*^2^) = 0.042	H-atom parameters constrained
*S* = 1.01	*w* = 1/[σ^2^(*F*_o_^2^) + (0.020*P*)^2^ + 1.2*P*] where *P* = (*F*_o_^2^ + 2*F*_c_^2^)/3
2402 reflections	(Δ/σ)_max_ < 0.001
151 parameters	Δρ_max_ = 0.38 e Å^−^^3^
0 restraints	Δρ_min_ = −0.50 e Å^−^^3^

### Fractional atomic coordinates and isotropic or equivalent isotropic displacement parameters (Å^2^)

**Table:**

	*x*	*y*	*z*	*U*_iso_*/*U*_eq_	
Pb1	1.0000	0.51034 (2)	0.7500	0.03248 (6)	
O1	0.99708 (9)	0.2668 (3)	0.85225 (9)	0.0427 (4)	
O2	0.92275 (9)	0.1874 (3)	0.75719 (9)	0.0442 (4)	
N1	0.88014 (11)	0.6287 (4)	0.64774 (11)	0.0418 (5)	
C1	0.92142 (13)	−0.0604 (5)	0.85353 (14)	0.0353 (5)	
C2	0.86958 (12)	−0.2024 (5)	0.81811 (13)	0.0363 (5)	
H2	0.8510	−0.1701	0.7725	0.044*	
C3	0.84468 (13)	−0.3932 (5)	0.84970 (14)	0.0402 (6)	
C4	0.87334 (17)	−0.4373 (6)	0.91770 (17)	0.0499 (7)	
H4A	0.8574	−0.5645	0.9395	0.060*	
C5	0.92466 (16)	−0.2977 (6)	0.95346 (14)	0.0562 (8)	
H5	0.9432	−0.3309	0.9991	0.067*	
C6	0.94919 (15)	−0.1064 (6)	0.92180 (14)	0.0499 (7)	
H6	0.9838	−0.0103	0.9461	0.060*	
C7	0.94876 (12)	0.1458 (5)	0.81944 (13)	0.0350 (5)	
C8	0.78855 (17)	−0.5504 (6)	0.8108 (2)	0.0580 (9)	
H8A	0.7464	−0.4889	0.8162	0.087*	
H8B	0.7946	−0.7127	0.8279	0.087*	
H8C	0.7890	−0.5497	0.7634	0.087*	
C9	0.82897 (17)	0.4775 (5)	0.62681 (17)	0.0474 (7)	
H9	0.8279	0.3363	0.6514	0.057*	
C10	0.77682 (16)	0.5196 (4)	0.56991 (16)	0.0443 (7)	
H10	0.7418	0.4095	0.5582	0.053*	
C11	0.77707 (11)	0.7242 (4)	0.53090 (11)	0.0303 (5)	
C12	0.83066 (13)	0.8809 (5)	0.55292 (14)	0.0415 (6)	
H12A	0.8335	1.0222	0.5289	0.050*	
C13	0.87981 (13)	0.8270 (5)	0.61065 (14)	0.0479 (7)	
H13	0.9148	0.9365	0.6243	0.058*	

### Atomic displacement parameters (Å^2^)

**Table:**

	*U*^11^	*U*^22^	*U*^33^	*U*^12^	*U*^13^	*U*^23^
Pb1	0.03302 (8)	0.03156 (8)	0.03056 (8)	0.000	0.00275 (5)	0.000
O1	0.0416 (10)	0.0463 (10)	0.0363 (10)	−0.0058 (8)	0.0017 (8)	0.0017 (8)
O2	0.0468 (10)	0.0512 (11)	0.0305 (9)	−0.0093 (9)	0.0010 (8)	0.0076 (8)
N1	0.0401 (12)	0.0448 (13)	0.0352 (12)	0.0023 (10)	−0.0016 (10)	−0.0019 (10)
C1	0.0374 (14)	0.0366 (12)	0.0334 (14)	0.0039 (10)	0.0112 (11)	0.0012 (11)
C2	0.0351 (13)	0.0412 (13)	0.0337 (13)	0.0056 (10)	0.0098 (10)	0.0021 (11)
C3	0.0400 (14)	0.0357 (13)	0.0472 (17)	0.0027 (11)	0.0150 (12)	0.0000 (12)
C4	0.0582 (19)	0.0490 (15)	0.0490 (18)	0.0026 (14)	0.0259 (15)	0.0110 (14)
C5	0.070 (2)	0.066 (2)	0.0322 (15)	0.0006 (16)	0.0110 (14)	0.0126 (14)
C6	0.0557 (18)	0.0589 (18)	0.0319 (15)	−0.0079 (15)	0.0036 (13)	0.0035 (14)
C7	0.0341 (13)	0.0398 (13)	0.0322 (13)	0.0032 (10)	0.0102 (11)	0.0027 (11)
C8	0.0487 (18)	0.0537 (17)	0.071 (2)	−0.0113 (14)	0.0126 (17)	0.0047 (17)
C9	0.0530 (18)	0.0427 (16)	0.0399 (16)	−0.0018 (12)	−0.0024 (13)	0.0074 (12)
C10	0.0450 (16)	0.0407 (15)	0.0400 (16)	−0.0107 (11)	−0.0044 (12)	0.0043 (11)
C11	0.0302 (12)	0.0331 (12)	0.0270 (12)	0.0007 (9)	0.0058 (9)	−0.0027 (10)
C12	0.0372 (14)	0.0412 (14)	0.0408 (15)	−0.0071 (11)	−0.0014 (11)	0.0059 (12)
C13	0.0374 (15)	0.0525 (16)	0.0470 (16)	−0.0086 (12)	−0.0040 (12)	−0.0014 (14)

### Geometric parameters (Å, °)

**Table:**

Pb1—O1	2.4803 (19)	C5—C6	1.391 (4)
Pb1—O2	2.4148 (19)	C5—H5	0.9300
Pb1—N1	2.893 (2)	C6—H6	0.9300
O1—C7	1.250 (3)	C8—H8A	0.9600
O2—C7	1.268 (3)	C8—H8B	0.9600
N1—C13	1.328 (4)	C8—H8C	0.9600
N1—C9	1.331 (4)	C9—C10	1.395 (4)
C1—C2	1.380 (4)	C9—H9	0.9300
C1—C6	1.388 (4)	C10—C11	1.381 (3)
C1—C7	1.507 (4)	C10—H10	0.9300
C2—C3	1.391 (4)	C11—C12	1.389 (3)
C2—H2	0.9300	C11—C11^i^	1.492 (4)
C3—C4	1.385 (4)	C12—C13	1.385 (3)
C3—C8	1.509 (4)	C12—H12A	0.9300
C4—C5	1.368 (4)	C13—H13	0.9300
C4—H4A	0.9300		
			
O2—Pb1—O2^ii^	84.54 (10)	C3—C4—H4A	119.3
O2—Pb1—O1^ii^	77.99 (7)	C4—C5—C6	120.2 (3)
O2^ii^—Pb1—O1^ii^	53.41 (5)	C4—C5—H5	119.9
O2—Pb1—O1	53.41 (6)	C6—C5—H5	119.9
O2^ii^—Pb1—O1	77.99 (6)	C1—C6—C5	119.3 (3)
O1^ii^—Pb1—O1	114.17 (9)	C1—C6—H6	120.4
O2—Pb1—C7^ii^	79.93 (7)	C5—C6—H6	120.4
O2^ii^—Pb1—C7^ii^	26.88 (6)	O1—C7—O2	121.8 (2)
O1^ii^—Pb1—C7^ii^	26.53 (6)	O1—C7—C1	119.8 (2)
O1—Pb1—C7^ii^	96.40 (7)	O2—C7—C1	118.4 (2)
O2—Pb1—N1	75.57 (7)	C3—C8—H8A	109.5
O2^ii^—Pb1—N1	125.75 (6)	C3—C8—H8B	109.5
O1^ii^—Pb1—N1	73.10 (6)	H8A—C8—H8B	109.5
O1—Pb1—N1	122.47 (6)	C3—C8—H8C	109.5
C7^ii^—Pb1—N1	99.23 (7)	H8A—C8—H8C	109.5
C7—O1—Pb1	91.10 (15)	H8B—C8—H8C	109.5
C7—O2—Pb1	93.70 (15)	N1—C9—C10	123.5 (3)
C13—N1—C9	116.1 (2)	N1—C9—H9	118.2
C13—N1—Pb1	118.81 (17)	C10—C9—H9	118.2
C9—N1—Pb1	124.04 (18)	C11—C10—C9	120.2 (2)
C2—C1—C6	119.9 (3)	C11—C10—H10	119.9
C2—C1—C7	121.2 (2)	C9—C10—H10	119.9
C6—C1—C7	118.9 (3)	C10—C11—C12	115.9 (2)
C1—C2—C3	121.0 (2)	C10—C11—C11^i^	122.3 (3)
C1—C2—H2	119.5	C12—C11—C11^i^	121.8 (3)
C3—C2—H2	119.5	C13—C12—C11	120.1 (3)
C4—C3—C2	118.2 (3)	C13—C12—H12A	120.0
C4—C3—C8	120.7 (3)	C11—C12—H12A	120.0
C2—C3—C8	121.0 (3)	N1—C13—C12	124.1 (3)
C5—C4—C3	121.4 (3)	N1—C13—H13	118.0
C5—C4—H4A	119.3	C12—C13—H13	118.0
			
O2—Pb1—O1—C7	0.62 (14)	C2—C3—C4—C5	0.2 (4)
O2^ii^—Pb1—O1—C7	−91.49 (15)	C8—C3—C4—C5	179.4 (3)
O1^ii^—Pb1—O1—C7	−51.27 (13)	C3—C4—C5—C6	0.1 (5)
C7^ii^—Pb1—O1—C7	−71.79 (18)	C2—C1—C6—C5	0.7 (4)
N1—Pb1—O1—C7	33.44 (17)	C7—C1—C6—C5	−179.4 (3)
O2^ii^—Pb1—O2—C7	78.48 (14)	C4—C5—C6—C1	−0.6 (5)
O1^ii^—Pb1—O2—C7	132.18 (16)	Pb1—O1—C7—O2	−1.1 (2)
O1—Pb1—O2—C7	−0.61 (14)	Pb1—O1—C7—C1	177.1 (2)
C7^ii^—Pb1—O2—C7	105.22 (16)	Pb1—O2—C7—O1	1.1 (3)
N1—Pb1—O2—C7	−152.43 (16)	Pb1—O2—C7—C1	−177.1 (2)
O2—Pb1—N1—C13	−179.6 (2)	C2—C1—C7—O1	−177.9 (2)
O2^ii^—Pb1—N1—C13	−107.4 (2)	C6—C1—C7—O1	2.2 (4)
O1^ii^—Pb1—N1—C13	−98.0 (2)	C2—C1—C7—O2	0.4 (4)
O1—Pb1—N1—C13	153.72 (19)	C6—C1—C7—O2	−179.5 (3)
C7^ii^—Pb1—N1—C13	−102.6 (2)	C13—N1—C9—C10	−0.6 (5)
O2—Pb1—N1—C9	−11.7 (2)	Pb1—N1—C9—C10	−168.7 (2)
O2^ii^—Pb1—N1—C9	60.4 (3)	N1—C9—C10—C11	1.5 (5)
O1^ii^—Pb1—N1—C9	69.9 (2)	C9—C10—C11—C12	−1.3 (4)
O1—Pb1—N1—C9	−38.4 (3)	C9—C10—C11—C11^i^	179.2 (3)
C7^ii^—Pb1—N1—C9	65.3 (2)	C10—C11—C12—C13	0.3 (4)
C6—C1—C2—C3	−0.4 (4)	C11^i^—C11—C12—C13	179.8 (3)
C7—C1—C2—C3	179.7 (2)	C9—N1—C13—C12	−0.5 (4)
C1—C2—C3—C4	−0.1 (4)	Pb1—N1—C13—C12	168.3 (2)
C1—C2—C3—C8	−179.3 (3)	C11—C12—C13—N1	0.7 (5)

Symmetry codes: (i) −*x*+3/2, −*y*+3/2, −*z*+1; (ii) −*x*+2, *y*, −*z*+3/2.

### Hydrogen-bond geometry (Å, °)

**Table:**

*D*—H···*A*	*D*—H	H···*A*	*D*···*A*	*D*—H···*A*
C13—H13···O1^iii^	0.93	2.54	3.461 (4)	172

Symmetry codes: (iii) −*x*+2, *y*+1, −*z*+3/2.
